# A comprehensive overview of diagnosis, imaging and treatment of vitreoretinal lymphoma

**DOI:** 10.1177/11206721231211931

**Published:** 2023-11-13

**Authors:** Matteo Menean, Chiara Giuffrè, Maria Vittoria Cicinelli, Alessandro Marchese, Giulio Modorati, Francesco Bandello, Elisabetta Miserocchi

**Affiliations:** 1 School of Medicine, Vita-Salute San Raffaele University, Milan, Italy; 2 Division of head and neck, Ophthalmology Unit, IRCCS San Raffaele Scientific Institute, Milan, Italy

**Keywords:** Vitreoretinal lymphoma, uveitis, multimodal imaging, primary central nervous system lymphoma, intravitreal chemotherapy, diagnostic vitrectomy, MYD88

## Abstract

Vitreoretinal lymphoma (VRL) is a rare B-cell intraocular neoplasia characterized by poor long-term prognosis and lack of effective therapies. It mainly involves the vitreous humor, the retina, and the retinal pigment epithelium (RPE), although anterior segment involvement can occur. VRL is classified as a lymphoma of immune privileged sites, along with testis lymphoma and primary central nervous system lymphoma (PCNSL). VRL and PCNSL are strictly connected indeed: 80% of VRL develop PCNSL, while 20% of patients with PCNSL present VRL during natural history of lymphoma. Due to the lack of worldwide consensus about diagnosis, therapy, and follow-up timing, VRL represents one of the most challenging ocular affections.

VRL commonly masquerades as a posterior uveitis, and misdiagnosis often occurs because of partial response to topical steroids. Gold standard for diagnosis is cytological analysis of vitreous humor. However, this technique lacks sensitivity and supplemental molecular analyses can improve the diagnostic process. Multimodal imaging allows ophthalmologists to empower their clinical suspicion and a comprehensive examination can highlight typical features of VRL and justify further invasive procedures.

There is no consensus about VRL therapy, and none of the therapeutical scheme has demonstrated to prevent cerebral involvement and improve patient's overall survival. Intravitreal injections of chemotherapeutics drugs, ocular radiation therapy and systemic chemotherapy can be considered in the treatment of VRL. Once cerebral involvement occurs, systemic chemotherapy must be included in the treatment as a life-saving therapy. Further multicentric studies are required to find out the best treatment of patients with VRL.

## Introduction and epidemiology

Vitreoretinal lymphoma is a rare B-cell malignant neoplasia, characterized by poor long-term overall survival due to CNS involvement. VRL potentially affects all ocular structures, but vitreous humor, RPE and retinal layers are most frequently involved.^1–6^ The 5th edition of the World Health Organization Classification of Hematolymphoid Tumors recently classified VRL in the group of lymphomas of immune privileged sites, along with testis lymphoma and primary central nervous system lymphoma (PCNSL).^7^ VRL and PCNSL are strictly connected: 80% of VRL develop PCNSL, while 20% of patients with PCNSL present VRL during natural history of lymphoma. VRL is commoly considered a primary lymohoma of the vitreous and the retina, despite the presence of PCNSL. Nonetheless, VRL rarely presents as a localization of a systemic lymphoma and in this case, it is defined as a secondary vitreoretinal lymphoma.^8^ The incidence of VRL has not yet been clearly quantified, due to the lack of world-wide multicenter studies, the rarity of the disease and the high rate of misdiagnosis. An approximate incidence varies from 0.027/100 000 person-year to 0.047/100 000 person-year.^9–12^ Recent statistics present even much higher incidence rate (1/100 000 person-year), mainly due to increased diagnostic suspicion and better diagnostic tools. VRL mainly affect patients in the sixth/seventh decade of life, although few cases have been reported in young immune-compromised patients. VRL equally affect male and females, without race predilection.^13–17^

## Etiology and pathogenesis

Little is known about VRL pathogenesis because of the low number of cytological specimens. However, VRL and PCNSL share many features and we could speculate a similar etiopathogenesis occurs.^1,4^ VRL rarely involves sites other than central nervous system (CNS) and testis. Retina, CNS and testis are immunological sanctuaries indeed, and lymphomas affecting these sites have been all grouped together by the 5th edition of the WHO Classification of Hematolymphoid Tumors in the group of lymphomas of immune privileged sites.^4,18^

There are two major theories on how VRL develops. The first speculates a malignant systemic B-cell migrates and homes into retina and vitreous humor. The second hypothesizes an inflammatory B-cell locates in the eye and acquires driving DNA mutations, with a speculative role of Epstein-Barr Virus (EBV) or Toxoplasma gondii.^19,20^ The homing process in the retina structure is crucial: of note, malignant B-cells of VRL ectopically present receptors (CXCR5 and CXCR4) for RPE-chemokines (CXCL13 and CXCL12, respectively).^21^

VRL neoplastic cells test positive for pan B-cell markers (CD20 and CD79a) at immunostaining. Conversely, they test negative at CD3 immunostaining, considered a pan T-cell marker.^4^ MYD88 l265P mutation has been frequently reported in VRL samples. MYD88 gene encodes for a protein involved in toll-like receptor signaling and its mutation could enhance cell proliferation and immortalization.^22–24^ In addition, a remarkable percentage of VRL samples showed mutations in CD79b encoding gene. CD79b is involved in BCR signaling can contribute to Nf-kb pathway hyperactivation.^23,25^

VRL sample immunoglobulin sequencing disclosed high rate of IGHV4-34 gene recurrence (64%), suggesting that antigen selection is part of etiopathogenesis of VRL.^26^

## Clinical features

VRL often masquerades as an intermediate or posterior uveitis, and mild response to topical steroid therapy is confounding and partially explain the diagnostic delay. Anterior can also be involved. In addition, a heterogeneous and unspecific plethora of ocular manifestations can occur in VRL.^1,27^

Most patients complain about hazy vision and floaters than can be misdiagnosed with posterior vitreous detachment, and these symptoms often remain underestimated for several months. Few patients require ophthalmological examination for severe decrease in visual acuity. At first evaluation, a diagnosis of idiopathic posterior uveitis is made, and topical steroid therapy is set up.^1,28^ Medium time from symptom onset to diagnosis ranges from 18 to 24 months.^28^

About half of the examined eyes present granulomatous or non-granulomatous keratic precipitates at anterior segment examination. Anterior chamber cells and flare can be disclosed as well. Occasionally, pseudohypopyon and iris/angle infiltration have been reported. Intermediate and posterior masquerading findings include variably severe vitritis, with clustering of inflammatory and neoplastic cells in strands, clumps and sheets along vitreous fibrils. Retinal signs of lymphomatous involvement are often unspecific. Most frequently, cream-colored yellow infiltrates can be observed. Although not frequently disclosed, pin-point yellow retinal lesions are quite typical of lymphomatous retinal infiltration. A mass-like appearance of retinal lymphomatous lesion has been also reported. Retinitis- or serpiginous-like appearance at fundus examination are rare but possible findings in eyes with VRL. Although unspecific, sings of vasculitis, fibrosis, RPE atrophy and optic nerve edema have been reported. Fundus examination should be performed carefully, with a focus on possible peripheral findings of inflammation, namely snowbanks and snowballs.^2,29–31^

Ophthalmoscopic examination should be supplemented with multimodal imaging techniques to empower diagnostic suspicion.^32^

## Multimodal imaging

A comprehensive analysis of posterior uveitis through multimodal imaging examination, comprehensive of color fundus photography, fundus autofluorescence (FAF), optical coherence tomography (OCT), fluorescein angiography (FA) and indocyanine green angiography (ICGA) can better characterize VRL imaging features and empower clinical suspicion.

### Fundus photography

**Figure 1.**
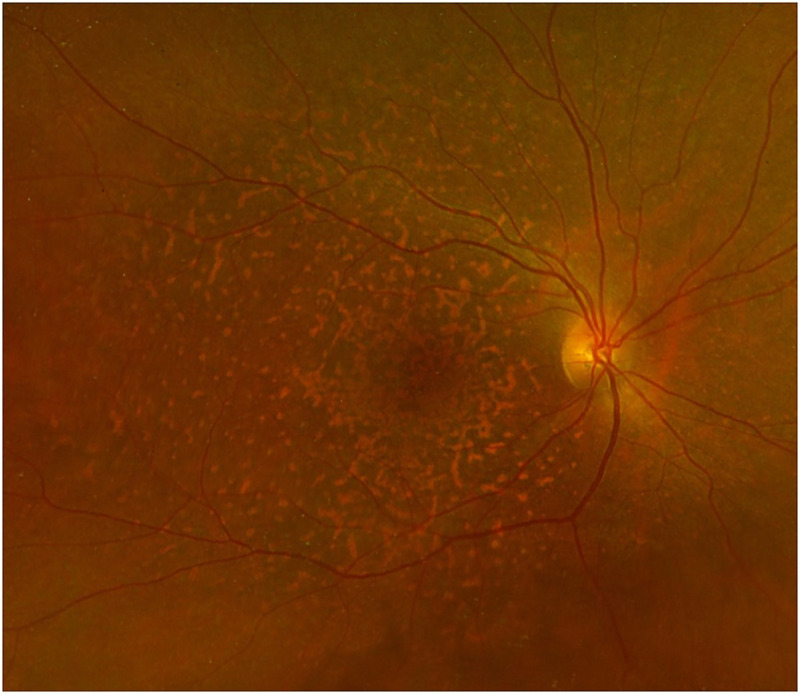
Multicolor fundus photography of a biopsy-proven VRL. Yellowish diffuse and irregular infiltrates can be disclosed at posterior pole.

Vitritis is the most common finding at fundus photography and can be disclosed in more than 90% of eyes. Leopard spot appearance can be rarely observed. (Figure 1) Seldom, yellow infiltrates at posterior pole or in the retinal periphery have been observed.^32–34^

### Fundus autofluorescence

**Figure 2.**
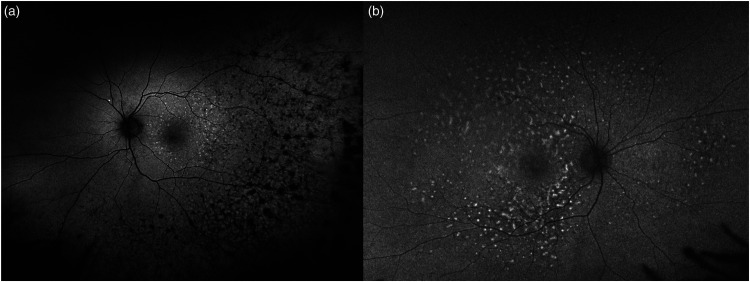
Ultra-widefield Fundus autofluorescence of two biopsy-proven VRL (a and b). Granular and mottled changes of alternating hyper- and hypo-autofluorescence can be disclosed in the retinal temporal mid-periphery (a) and at posterior pole (b). A leopard skin like appearance can be observed (b).

Fundus autofluorescence has high sensitivity and detects autofluorescence changes in a large percentage of cases. Particularly, alternating hypo- and hyper-autofluorescent lesions can be observed. Lymphomatous infiltration causes a masking effect, reducing the light absorption and emission. Conversely, inflammatory reaction and activation of RPE and retinal atrophy hesitate in hyper-autofluorescence. These features can be combined, resulting in reticular or leopard skin appearance. (Figure 2).^29,32–36^

### Optical coherence tomography

**Figure 3.**
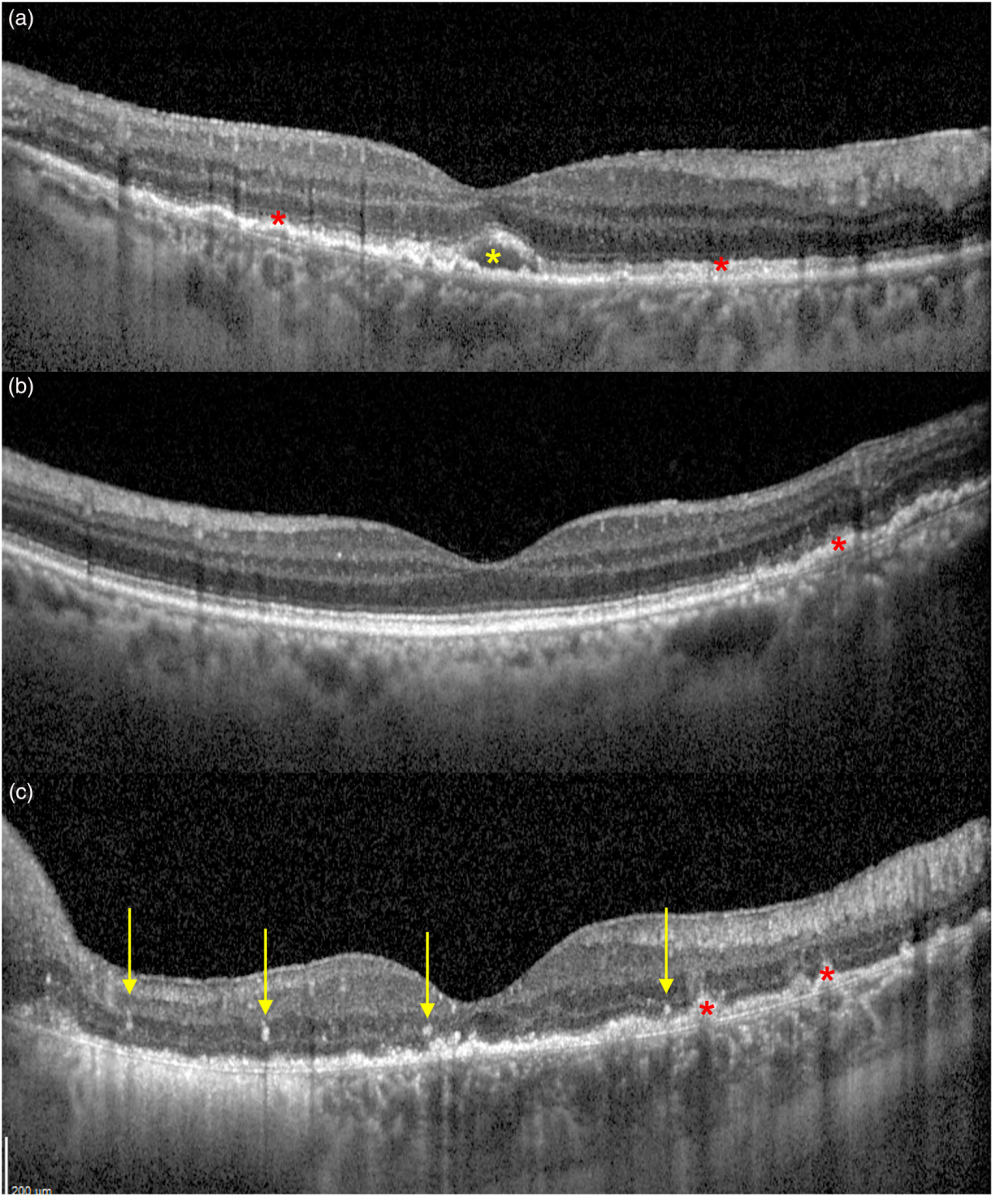
B-scan Optical coherence tomography (OCT) of three biopsy-proven VRL. Retinal pigment epithelium (RPE) thickening and mottling can be disclosed, as well as small sub-RPE deposits (a, b and c, red asterisks). A small macular subretinal hypo-reflective deposit is marked in A (yellow asterisk). Vertical hyper-reflective spots are signs of intraretinal lymphomatous infiltration and have been highlighted in C (yellow arrows).

Since VRL can virtually affect all retinal layers, the vitreous humor and the RPE, a huge heterogeneity of OCT features can be observed in eyes with VRL. (Figure 3) Vitreous opacities are frequently disclosed at OCT and can hesitate in low-quality imaging. Preretinal and intraretinal hyperreflective deposits can be observed in less than a half of patients. Intraretinal deposits can be diffuse or can present as discrete hyperreflective vertical lesions. Focal or diffuse subretinal deposits can be seldom disclosed as well, with high variability in size and location. RPE changes are frequently reported in eyes with VRL, with granular or mottling changes, always confirmed through a granular appearance at FA. Sub-RPE deposits are a common and typical OCT-feature of VRL and are usually characterized by a hyper-reflective content. Of note, VRL can affect retinal periphery without macular involvement, and OCT scans can eventually report no signs of retinal involvement.^32,37–40^

### Fluorescein angiography

Lymphomatous and inflammatory retinal infiltration and RPE changes (i.e., RPE mottling) often present as hypo-fluorescent spots at late phase FA. These findings can frequently be observed in the retinal periphery, and the posterior pole can be completely spared.

**Figure 4.**
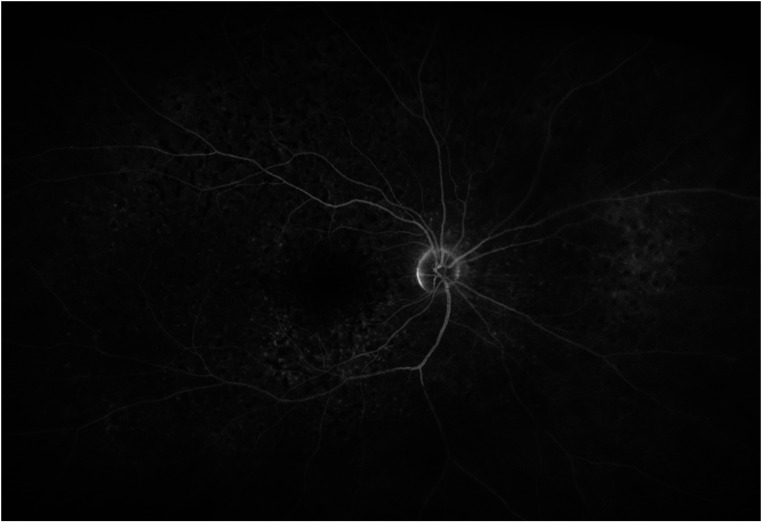
Late phase fluorescein angiography of a biopsy-proven VRL. Diffused alternating areas of hyper- and hypo-fluorescence can be observed, with a leopard spot appearance.

Discrete window defects can be disclosed as well as punctate hyperfluorescent spots. RPE changes and subretinal infiltration can produce a staining effect at FA, with limited or punctate areas of hyper-fluorescence. All these characteristics frequently overlap, with a granular alternating hyper/hypo-fluorescent appearance, with a leopard spot appearance. (Figure 4).^32–34,36^

### Indocyanine green angiography

VRL does not affect choroid. At ICGA, discrete hypo-fluorescent lesions can be detected at the posterior pole and are due to masking effect of lymphomatous retinal infiltration.^32^

## Diagnosis

Although ophthalmoscopic examination and multimodal imaging characterization confirm the clinical suspicion, cytopathological and immunohistochemical examination of ocular specimen is still the diagnostic gold standard for VRL.^41^ Particularly, the detection of malignant clonally transformed B-cells is required perform VRL diagnosis.^42,43^ To achieve this purpose, vitreous biopsy though trans pars plana vitrectomy (TPPV) is the procedure most frequently performed. On the other hand, the use of chorioretinal biopsy is restricted to complex or dubious cases. Since vitritis is a common finding in eyes with VRL, TPPV often has a therapeutic purpose. During TPPV, low vitrector cut rate (600 cuts per minute), possibly under air infusion, is recommended to avoid cell damage and improve diagnostic sensitivity. Vitreous humor samples must be undiluted for cytopathological analysis, and immediately transferred to the laboratory without fixation. As an alternative, mild fixative agents (Cytolyt or HOPE fixation) could be used. Formalin fixation cannot be considered as an alternative and must be avoided, in order to preserve cell morphology and immunoreactivity.^44–47^ Despite these efforts, diagnosis is still challenging due to the low number of neoplastic lymphocytes, previous treatments of corticosteroids and the fragile nature of the specimen. Consequently, there is a large percentage of false-negative vitreous biopsies.

Both in the aqueous and in the vitreous humor, a specific VRL-associated cytokine profile has been disclosed, particularly with high levels of IL-10. In fact, B cells produce IL-10 and high levels are due to uncontrolled B-cell proliferation. Nevertheless, a commonly defined threshold for IL-10 levels has not been established.^23,48–50^ In addition, IL-10/IL-6 ratio is thought to be more sensitive in VRL detection. IL-6 is physiologically produced during non-neoplastic inflammatory processes, and IL-10/IL-6 ratio helps ophthalmologists finding out masquerading syndromes. The positive and negative predictive values of IL-10/IL-6 ratio for VRL are 95% and 71%, respectively.^42,51,52^ However, low IL-10/IL-6 revels levels do not rule out the diagnosis of VRL. Costopoulos et al proposed a new score, the Interleukin Score for intra-Ocular Lymphoma Diagnosis (ISOLD), applied both on aqueous and vitreous samples. The ISOLD score has already undergone validation, making it a reliable diagnostic tool for distinguishing between lymphoma and uveitis.^53,54^ Immunocytological techniques and flow cytometry can phenotype examined cells based on their surface markers. Malignant B-cells present restricted expression of either the k or λ chain, positively staining for CD19, CD20 and CD22.^23,55^

According to recent mutational analyses of DLBCL and VRL specimens, MYD88 l265P mutation has been reported in a large percentage of examined samples.^56^ Additionally, Yonese et al. showed that 35% of VRL vitreous samples presented CD79b mutations.^57^ The MYD88 l265P mutation has also been identified as a potential diagnostic target using aqueous tap. Aqueous tap has been suggested as an adjuvant and complementary diagnostic technique for early diagnosis of PVRL since it is a complementary procedure compared to diagnostic vitrectomy.^24^

Whenever PCNSL is suspected, cerebrospinal fluid (CSF) evaluation should be performed. Approximately 25% of individuals with MRI-visible lesions will also have positive CSF cytology results. To rule out additional infectious and non-infectious uveitis causes, extensive blood tests should be done. EBV and HIV serology and a complete blood count are helpful to extensively evaluate patient's systemic clinical status.^58,59^

## Treatment

The gold standard treatment for VRL has not yet been established. Several intraocular and systemic therapeutical options are available, although none of them has demonstrated its efficacy in eradicating lymphoma and preventing disease progression.^4^ CNS involvement accounts for poor prognosis of VRL and represents a key factor in determining therapies. Consequently, gadolinium-based MRI is highly recommended immediately after VRL diagnosis and periodically, to detect early signs of cerebral lymphomatous involvement.

In case of isolated VRL, without signs of cerebral lymphomatous involvement, treatment goals are both controlling and eradicating VRL and preventing CNS involvement. However, despite therapeutic efforts, VRL disseminates to the brain in 60–90% of patients.^4^ Since local therapies can only control the intraocular burden of VRL, without preventing CNS involvement, systemic prophylactic chemotherapies have been considered as an option. However, they have not demonstrated their efficacy and a global consensus is still lacking.^4,5^

De la Fuente et al prevented brain lymphomatous dissemination treating patients with VRL with bilateral radiation therapy and systemic methotrexate: they reported lower incidence rates of CNS involvement, compared to other studies (37.5% vs 56–85%).^1,28,60^ The justification for this approach is that many VRL patients may have latent CNS involvement that is not detectable and cannot be treated with intraocular therapies alone; instead, they need systemic chemotherapy to prevent CNS lymphoma.^61^ However, main weaknesses of this data are due to the lack of multicenter randomized trials.

In other studies, the progression free survival, but not the overall survival, was significantly improved when intravitreal and systemic chemotherapies were combined.^1^ However, Riemens et al. did not demonstrate that this combined approach was superior to local therapy alone.^42^ Hormigo et al. reported that VRL treated with preventative chemotherapy and/or radiotherapy had a considerably higher median survival rate than the group treated after CNS involvement was diagnosed.^5^ The International Primary CNS Lymphoma Collaborative Group recommended high dosage of systemic chemotherapy, combined with intravitreal chemotherapies or ocular radiation therapies for isolated VRL.^51^ However, Hashida et al. demonstrated prophylactic systemic chemotherapy significantly delays but not prevents the onset of cerebral involvement.^62,63^

Local therapies include intravitreal injection of chemotherapeutic drugs and ocular radiation therapy. There is no evidence one choice is better than the other one. The decision should be taken based on disease's laterality, the patient's preferences and other relevant practical factors. For patients with VRL without CNS involvement, external-beam radiation (EBRT) has been suggested. It delivers a total of 35 to 40 Gy to both eyes in 15 fractions of 2 Gy each using opposing lateral beams. Recurrence and radiation retinopathy rates have been rarely reported with this dosage. Cataract is a frequent complication of ocular radiation therapy and is easily surgically managed.^4,29,64^

Intravitreal therapies deliver drugs in the vitreous humor through an injection. Many regimens of methotrexate and rituximab injections have been proposed in VRL management. The most used treatment scheme consists of intravitreal methotrexate (400 μg in 0.1 ml) twice weekly for four weeks, once weekly for eight weeks during the consolidation phase, and once a month during the maintenance phase for nine months. Recurrences with this course of treatment have been demonstrated to be extremely uncommon, and only a few side effects, such as corneal epitheliopathy and a brief increase in intraocular pressure, have been reported.^65^ Intravitreal administration of rituximab (1 mg in 0.1 ml) for four weeks is considered a valid alternative.^66^ A lower frequency of intravitreal injection allows lower risk of corneal toxicity, despite preserved therapeutic efficacy.^29,66^

Systemic therapies comprehend induction and consolidation phase and are the gold standard treatment when cerebral involvement has been proven.^59,67–69^ Induction therapy is based on high dose methotrexate. Remission rate of PCNSL and VRL scored 72% and 94%, respectively. According to the International Extranodal Lymphoma Study Group (IELSG) 32 trial, the combination of methotrexate, cytarabine, thiotepa and rituximab (MATRix) is considered the gold standard in fit patients aged less than 70 years. The MATRix regimen could be combined to whole brain radiation therapy, although the high rate of adverse events should be considered. Sixty percent (60%) of patients typically experience a full response after induction chemotherapy.^70^ Nonetheless, they still require consolidation therapy to minimize the risk of disease relapse. Whole brain radiation therapy, systemic chemotherapies, or autologous stem cell transplant represent different possibilities of consolidation.^59,70^

In case of PCNSL relapse, several treatments are available. If a positive response during the initial treatment phase was observed, it's conceivable to re-administer HD-MTX.^59^ Other alternatives encompass thiotepa-based chemotherapy followed by ASCT, intrathecal cytarabine, high dose cytarabine and pemetrexed, along with lenalidomide, pomalidomide, or ibrutinib.^59^ Particularly, lenalidomide and temozolomide showed encouraging results as single agent oral therapies, and further studies are required to explore their effectiveness as first-line option.^71,72^ Similarly, ibrutinib has been extensively investigated as a viable choice for relapsing and refractory PCNSL and VRL, due to the inhibition of BTK, an enzyme implicated in BCR signaling and lymphoma proliferation, and showed clinical activity in both phase I and phase II studies.^73^

## Prognosis

Poor prognosis of patients with VRL is due to cerebral involvement, and 5-years mortality rate can reach 90%.^15,74,75^ As a matter of fact, CNS involvement is the worst prognostic factor in patients with VRL.

Sub-RPE infiltrates have been proposed as OCT biomarkers of poor prognosis for overall survival, progression free survival and visual outcome, although statistical significance has not been reached.^69,76,77^

The IELSG score is the most important and validated prognostic score for patients with PCNSL: therefore, it is applicable to those patients with both VRL and cerebral involvement. This score includes systemic, laboratory and radiologic parameters, namely lactate dehydrogenase (LDH) levels, CSF proteins levels, lymphoma localization (cerebellum, basal ganglia, brainstem, periventricular zone), patient's age and performance status, according to the ECOG classification.^69,77^

In conclusion, CNS involvement represent the worst ocular and overall survival negative prognostic factor, with a range of medium survival time from 1 to 3 years, according to different reports.^1,75^

## Conclusion

VRL represents the most frequent underlying cause of masquerade syndrome. No univocal consensus has been established for diagnosis and treatment, and VRL still represents a challenge for ophthalmologists and oncologists-hematologists. In case of recurrent uveitis with poor response to steroids, patients should be promptly referred to tertiary referral centers to carry out a proper diagnosis. A complete ophthalmological imaging allows to highlight variably typical features of VRL, and FAF, OCT, FA and ICGA should be performed in case of prolonged posterior uveitis. Cytologic analysis of vitreous samples and immunohistochemistry represent the diagnostic gold standard for VRL, but low sensitivity justifies the use of supplemental molecular analysis, as MYD88 l265P mutation detection and IL-10 and IL-6 levels analyses. There is not worldwide consensus for the treatment of VRL. Although systemic chemotherapy is the primary option in patients with concurrent PCNSL, the best treatment for VRL without cerebral involvement has not been found. Particularly, none of the treatments have demonstrated to effectively prevent cerebral lymphomatous dissemination, and the debate is still open. Particularly, it is not clear whether systemic chemotherapy combined with intravitreal methotrexate can prevent VRL progression in PCNSL.

Prognostic markers at multimodal imaging have not been found, and the univocally recognized negative prognostic factor for overall survival is the CNS involvement.

Large multicentric clinical trials will be able to compare different treatment and to clarify the best therapeutic management for patients with VRL, although the rarity of the disease highly limits these studies and explains the low statistical significance of the presented data.
